# Behavioral and Metabolic Risk Factors for Noncommunicable Diseases among Population in the Republic of Srpska (Bosnia and Herzegovina)

**DOI:** 10.3390/healthcare11040483

**Published:** 2023-02-07

**Authors:** Aleksandar Majić, Daniela Arsenović, Dimitrije D. Čvokić

**Affiliations:** 1 Faculty of Natural Sciences and Mathematics, University of Banja Luka, 78000 Banja Luka, Bosnia and Herzegovina; 2 Faculty of Sciences, University of Novi Sad, 21000 Novi Sad, Serbia

**Keywords:** risk factors, population, noncommunicable diseases, Republic of Srpska

## Abstract

Noncommunicable diseases (NCDs) are the major cause of death worldwide, and they are attributable to genetic and physiological determinants, behavioral risk factors and environmental impacts. The aim of this study is to assess behavioral risk factors for metabolic disease using demographic and social–economic aspects of the population characterized by risk factors, and to investigate relations among lifestyle risk factors (alcohol consumption, tobacco use, physical inactivity, intake of vitamins, fruits and vegetables) that are responsible for the majority of NCD deaths in the Republic of Srpska’s (RS’s) population. This is a cross-sectional study based on the analysis of a survey conducted among 2311 adult (≥18 years) persons (54.0% women, and 46.0% men). The statistical analysis was carried out by using Cramer’s V values, clustering, logistic regression (binomial, multinomial and ordinal), a chi-square test and odds ratios. In the case of logistic regression, we provide the prediction accuracy in percentages. A significant statistical correlation between demographic characteristics (gender and age) and risk factors was observed. The highest difference according to gender was observed in alcohol consumption (odds ratio (OR) = 2.705, confidence interval (95% CI) = 2.206–3.317), particularly in frequent consumption (OR = 3.164, 95% CI = 2.664–3.758). The highest prevalence of high blood pressure was registered in the elderly (66.5%); the same holds for hypertension (44.3%). Additionally, physical inactivity was one of the most common risk factors (33.4% physically inactive respondents). A significant presence of risk factors was confirmed among the RS population, with higher involvement of metabolic risk factors among the older population, while the prevalence of behavioral factors was related to younger age groups, particularly in the case of alcohol consumption and smoking. A low level of preventive awareness was observed among the younger population. Therefore, prevention is one of the most important instruments related to decreasing NCD risk factors in the RS population.

## 1. Introduction

Noncommunicable diseases (NCDs) are the most common causes of long-term illness, disability and premature mortality worldwide [1,2] and disproportionately affect the population in low-income and low–middle-income countries [3]. According to the World Health Organization (WHO), NCDs were responsible for 74% of deaths, worldwide [4]. About 80% of the deaths occurred in low- and middle-income countries, and 25% occurred in people younger than 60 [5]. Among NCDs, leading causes of death are cardiovascular diseases (17.9 million deaths annually), cancer (9.0 million), respiratory diseases (3.9 million) and diabetes (1.6 million) [6,7]. These four causes of death together account for more than 80% of all premature NCD mortalities [8]. A considerable number of cases of NCD burden are attributable to behavioral, environmental, dietary and metabolic risk factors [9,10]. Factors identified as major determinants of NCD prevalence are raised blood pressure (hypertension) [11,12,13,14,15,16,17], raised total cholesterol (hyperlipidemia) [18,19,20,21], tobacco use [22,23,24], alcohol consumption [22,25,26,27,28], physical inactivity [29,30,31,32], unhealthy diet [33,34,35], overweight and obesity [36,37,38] and raised blood sugar (diabetes) [39,40,41,42,43]. Similar to these findings, Divajeva et al. (2014) indicated that most NCDs are caused by preventable risk factors [44].

Regardless of the fact that conditions related to NCDs are usually associated with older populations, all ages are at risk [7]. Evidence shows that more than 15 million of all deaths attributed to NCDs occur in ages from 30 to 69 years [45]. The results of the research conducted by Licher et al. [1], in which participants from the Rotterdam study were followed, suggest that, in Western European countries, nine out of ten persons aged 45 years or older develop an NCD during their remaining lifetime, while at least a third are, in addition, diagnosed with multiple NCDs.

Data from developed countries have shown that, in relation to NCDs, disadvantaged and marginalized individuals and communities are more vulnerable, compared to people with a higher socioeconomic status [46]. Recent studies [47,48] that examined socioeconomic inequalities in developed countries have found that a healthy life expectancy is far less favorable in lower socioeconomic groups. Additionally, it has been found that persons with higher levels of education not only live longer but also spend more years in better health than those with lower education [49]. Social gradients in risk factors account for more than half of the inequalities in major NCDs, particularly for cardiovascular diseases and lung cancer. People in low-income countries and those with a low socioeconomic status also experience more difficulty in accessing health services for timely diagnosis and treatment of NCDs than those in high-income countries or those with a higher socioeconomic status [10]. In this context, reducing socioeconomic inequalities could promote a healthy life expectancy and increase the life span in the overall population. NCDs generally precede the fatal outcome, which implies that a significant proportion of the population spend many years in poor health. Effective prevention measures can prolong individuals’ lives and significantly improve their quality of life [43]. Avoiding risk factors is called primordial prevention, which is crucial in reducing morbidity from NCDs [50].

Reports about NCDs in 2014 suggest that, among the major risk factors, smoking prevalence among males is the highest in Eastern and Central Europe [51]. Additionally, the highest NCD burden related to alcohol consumption was registered in this part of Europe [9]. The population in Eastern and Central Europe is characterized by the highest blood pressure levels, compared to high-income populations [52]. All analyzed lifestyle factors, together with demographic trends in population aging, bring substantial challenges for governments, policy makers and public health systems. Moreover, NCDs represent a great burden for the health system of the Republic of Srpska (RS), making up over 80% of the total mortality in 2019 [53]. According to the cause of death, most deaths are due to diseases of the circulatory system (I00–I99) (49.6%) and neoplasms (C00–D48) (19.2%).

The aim of the study is to assess behavioral risk factors for metabolic diseases, using demographic and social–economic (independent) variables as drivers of NCDs (dependent variables). Based on the obtained results, this study identifies the role and importance of risk factors that are recognized as the most common causes of morbidity and mortality in the Republic of Srpska’s (RS’s) population.

## 2. Materials and Methods

### 2.1. Study Design and Data

The RS is one of the two entities of Bosnia and Herzegovina, covering an area of 24,641 km^2^, with a population of 1,142,495 (estimated for 2019) [53]. Analysis of the demographic and social–economic aspects of the population characterized by risk factors of NCDs was conducted as a cross-sectional study using an anonymous survey based on a questionnaire. The survey was conducted in 36 larger cities and municipalities in the RS in August, September, and October 2019 (B and H). The selection of cities and municipalities was based on their demographic size. Surveys were carried out by the authors of this study, by going to the cities and municipalities.

The total number of people who participated in the survey was 2311. The inclusion criterion was adults 18 years and older of both sexes. Exclusion criteria applied to persons under 18 years of age, as the study was conducted on the adult population. Incomplete answers were not included in the analysis of the results, but the share of these questionnaires was minimal (below 0.1% of the sample). The survey was conducted in various institutions, economic entities, state and private companies, and service activities, within the framework of several scientific seminars, while the largest number of respondents were surveyed on public lands, using a random sampling method. Most participants were from the largest city, Banja Luka (45.4%). 

The sample respondents make up more than 0.2% of the population of the RS. Given that different categories of the population cover different geographical areas, the obtained results can be considered representative.

The questionnaire was compiled based on the methodology of wave 3 of the European Health Interview Survey (EHIS wave 3) [54] and the recommendations of the WHO [55]. The questionnaire contained 16 closed-ended questions related to demographic, geographical and socioeconomic characteristics, and the risk factors affecting the incidence of NCDs. Demographic and socioeconomic variables consisted of questions related to: gender (male, female), age (in years), type of settlement (urban, suburb, rural), level of education (primary, secondary, tertiary), working status (employed, unemployed, student, retired) and income (low, middle, high). 

This study covers the main lifestyle (smoking and tobacco use, alcohol consumption, frequent alcohol consumption, physical activity, intake of vitamins, fruits and vegetables) and metabolic risk factors (blood pressure levels, cholesterol levels, blood glucose levels, and overweight and obesity).

When asked about cholesterol, blood pressure and blood sugar levels, respondents answered yes, no or I don’t know. The limit values were determined on the basis of WHO recommendations [55] relating to persons over 18 years of age. The age-standardized prevalence of high blood pressure is ≥140/90 mmHg, and that of high total cholesterol is ≥5.0 mmol/L, while the age-standardized prevalence of high blood sugar is ≥7.0 mmol/L. 

The level of overweight and obesity was determined based on the body mass index (BMI). BMI was calculated based on the height and weight of the participants in the questionnaire. The age-standardized prevalence of overweight and obesity in persons aged 18+ years is defined as a body mass index of ≥25 kg/m^2^ for overweight and a body mass index of ≥30 kg/m^2^ for obesity.

Issues related to behavioral risk factors were related to smoking and tobacco use (daily smoker, non-daily smoker, never/ex-smoker), alcohol consumption (yes, no), physical activity longer than 150 min weekly (yes, no) and the intake of vitamins, fruits and vegetables (low, middle, high).

Questions related to frequent alcohol consumption were based on AUDIT-C questions (monthly or less, 2–4 times a month, 2–3 times a week, 4+ times a week) [56]. 

In the last question (How frequently do you check the blood count and preventively visit the doctor? (per year)), respondents gave the following answers: by no means, once, twice and repeatedly. The aim of this question was to examine the connection between demographic and socioeconomic variables with the degree of preventive awareness among the population of the RS.

### 2.2. Statistical Analysis

Statistical analysis was performed using the statistical package SPSS Statistics (IBM SPSS Statistics for Windows, Version 20.0. Armonk, NY, USA: IBM Corp.) and statistically oriented Python programming packages (libraries), such as pandas [57], statsmodels [58], scikit-learn [59] and kmodes [60]. For data visualization, we used the Python packages matplotlib [61] and seaborn [62]. 

The missing values were dealt with using four techniques: column elimination (for comparison), row elimination, imputation using mode values, and nearest neighbor imputation (KNN imputation). The results obtained using the aforementioned techniques were compared, to gain insight into how the missing data can affect our analysis and the corresponding inferences. The row elimination technique, depending on what is done with the data, affects 40 rows at most, which is about 1.74% of our data set; the row elimination technique is suitable in general because the data set reduction is insignificant.

Only questions about age contained data from the interval scale, while the statistical analysis of the majority of the questions was performed based on the nominal scale. Given the large representation of the nominal type of data, a chi-square test was used in the analysis, alongside Cramer’s V values. In particular, Cramer’s V correlation coefficients are given in the form of a heat map (Figure S1), to present the association strengths between sociodemographic and health variables more vividly.

In order to gain a better initial insight into our data set, we used *k*-mods. The *k*-mods algorithm was used because most variables are nominal. The best number of clusters was determined using the elbow method (Figure S2). The set of possible cluster quantities was taken to be {1, …, M}, where M represents the number of all variables that are of interest to us in this research. In our case, the M value was 21. The elbow method was repeated 100 times, and the median elbow value was taken as the optimal number of clusters. For the final initialization, Cao’s method [63] was utilized. The most interesting observations, regarding the clustering, are plotted as histograms.

Statistical analysis concerning smoking, alcohol consumption and physical inactivity was performed using binary logistic regression. Multinomial logistic regression was used in the statistical analysis focusing on the blood pressure level, cholesterol level and blood sugar level, while ordinal logistic regression was applied to the following target variables: overweight and obesity, intake of vitamins, fruits and vegetables, blood count test and health control. In the analysis, the missing values were tackled in all aforementioned applicable ways. The train and test split ratio took values from the set {0.20, 0.21, 0.22, 0.23, 0.24, 0.25, 0.26, 0.27, 0.28, 0.29, 0.30}. For each missing value technique and each split value, the computation was repeated 10 times. This approach resulted in 330 computations for each target variable. The accuracy score was used as a prediction metric. From the set of all computations for a particular target variable, we decided to present the median, regarding the accuracy score. The exception was the ordinal regression, as the prediction values are composed of fractions of correct values. Additionally, logistic regression was used in the statistical analysis between the three age groups (<35 age, 35≤ age <55 and ≥55 age).

The odds ratios (ORs) were also obtained for behavioral and metabolic risk factors in relation to the demographic and socioeconomic characteristics. The odds ratio analysis was conducted on the basis of a dichotomous division of independent variables into gender (male, female), age (from 18 to 44 years, over 45 years), type of settlement (urban, rural, and suburban), education level (tertiary, primary, and secondary), working status (employed, unemployed) and income (high and middle compared to low). In the case of the dependent variables (risk factors), grouping was performed on the basis of prevalence (answer to the questionnaire “yes”). Overweight and obesity were divided into >25 BMI and <25 BMI, and intake of vitamins, fruits and vegetables was divided into low, middle and high, while in the case of blood control, the grouping was as follows: respondents who do not check their blood count versus those who do.

The analysis was performed based on a statistical significance of *p* < 0.05 and a 95% confidence interval (95% CI).

## 3. Results

Out of the total number of respondents (2311), the structure by gender indicates a higher representation of women, at 54% (1249 respondents), compared to men, who accounted for 46% (1062 respondents). The average age of the respondents was 37.8 years. Analysis of the age and risk factors of the respondents was categorized into six age cohorts. About two thirds of the respondents were from urban settlements. A significantly smaller number of respondents were from suburban settlements and rural settlements. The largest number of respondents completed secondary education, while the smallest number of respondents have higher and primary education. More than half of the respondents were employed, followed by students, unemployed people and retirees (Table 1).

The structure of the respondents according to gender and work status (presented in Table 1) corresponds approximately to the total population of the Republic of Srpska. However, the biggest deviations of the total population and the structure of the respondents were registered in the type of settlement and, partially, in the age structure of the respondents. In the type of settlement, there was a noticeable deficit of the rural population, while the average age of the respondents was slightly lower than the structure of the population in the RS. Precisely because of the deficit of the old population, metabolic risk factors have more favorable tendencies in the RS than in neighboring countries.

In Figure S1, there are three quite distinguishable spatial clusters regarding higher Cramer V correlation coefficients. The fourth one comprises the low correlation values (the dark squares), which is also the largest cluster. In particular, the heat map of Cramer’s V coefficients in Figure S1 suggests interesting results and a statistically significant connection concerning the following:

- Gender and alcohol consumption;
- Age and hypertension, cholesterol and diabetes;
- Education level and hypertension;
- Employment and hypertension, cholesterol and diabetes.

This observation suggests that demographic factors such as age, education level and employment are the most relevant features. Furthermore, we can see that their connection with the set of metabolic traits is the most distinguishable, in comparison with the behavioral ones. Additionally, it seems that the type of settlement does not exacerbate any connection between health variables, which could be interesting by itself. 

The maximal Cramer V correlation coefficient is 0.47, regarding the connection between age and hypertension. The second highest coefficient corresponds to the pairs age–cholesterol, and employment–hypertension. Although the coefficients do not indicate a strong correlation, as none is larger than 0.7 (moreover, none is larger than 0.5), we point out that the size of the data set can affect the Cramer V coefficients, and that these values should be considered relative to each other. In other words, the heat map in Figure S1 essentially serves as guidance for the research roadmap.

One can observe that the number of clusters, determined by the elbow method, is neither large, nor small, i.e., it is quite reasonable. It is natural to try to find some direction for research and analysis by inspecting corresponding histograms.

In accordance with these observations, we found dominant characteristics in our clusters (Figure S3) regarding the following metabolic attributes: hypertension (Figure S3a), cholesterol (Figure S3b), and diabetes (Figure S3c). For all other features, we could not observe the dominance, nor the dominant characteristics, across our clusters. We believe that this, again, suggests that the focus of regression analysis should be on the metabolic features, regarding the target variables.

### 3.1. Gender and Risk Factors

Pronounced differences by gender were observed in two behavioral risk factors (Table 2). Regarding alcohol consumption, divergence was found in terms of frequent consumption (Table 3). The prevalence of alcohol consumption for men was significantly higher compared to women. More than 80% of the male population consumed alcohol, and almost half of the males consumed alcohol at least once a week (Table 2). Statistically significant differences by gender were also found for physical inactivity, with lower odds of physical inactivity associated with males (Table 4). For smoking and tobacco use as risk factors, there were no statistically significant differences by gender.

The prevalence of metabolic risk factors was more pronounced for men. High blood pressure was more frequent in men. The prevalence of diabetes was present in less than 10% of respondents for both genders, with a slightly higher value for men. Additionally, a greater number of respondents with overweight and obesity (BMI > 25) were registered among men (Table 4). The prevalence of cholesterol was slightly higher in women. Large differences between gender were observed in the intake of vitamins, fruits and vegetables. Low intake was found in a quarter of the male respondents. Health control is much more characteristic of the women. As many as 36.1% of the men do not go to health examinations at all, while the percentage for women is lower.

Statistically significant differences by gender were registered for blood pressure level, overweight and obesity, cholesterol level, and blood glucose level. Additionally, blood count tests and health control were more pronounced among the women than the men (Table 3), which is, interestingly, not observable from the Cramer V heat map and cluster visualizations. The binomial logistic regression revealed that gender, as a factor, has the biggest impact on blood pressure and physical inactivity (Table 5).

### 3.2. Age and Risk Factors

The most significant differences (*p* < 0.001) were observed between age contingents and high blood pressure, high cholesterol, elevated blood sugar, and overweight and obesity (Table 3). The most vulnerable group of respondents is the population over 65. About 2/3 of this population group had high blood pressure, more than 40% had high cholesterol levels and 26.9% had high sugar levels, while 35.9% of the elderly were overweight and obese (Table 3). Simultaneously, an extremely low prevalence of metabolic factors was observed in the 18–44 age group for hypertension, elevated cholesterol and diabetes (Table 4).

The average age for the occurrence of hypertension was 53.7 years. For males, it was 51.5 years of age, while for females, it was 56.0 years of age. The same holds for elevated cholesterol, which, on average, occurred in males at 51.2 years of age, while high cholesterol in females occurred at 55.3 years of age. Female respondents who had diabetes were, on average, 52.6 years of age, while males with this disease were, on average, 54.6 years of age. 

**Figure 1:**
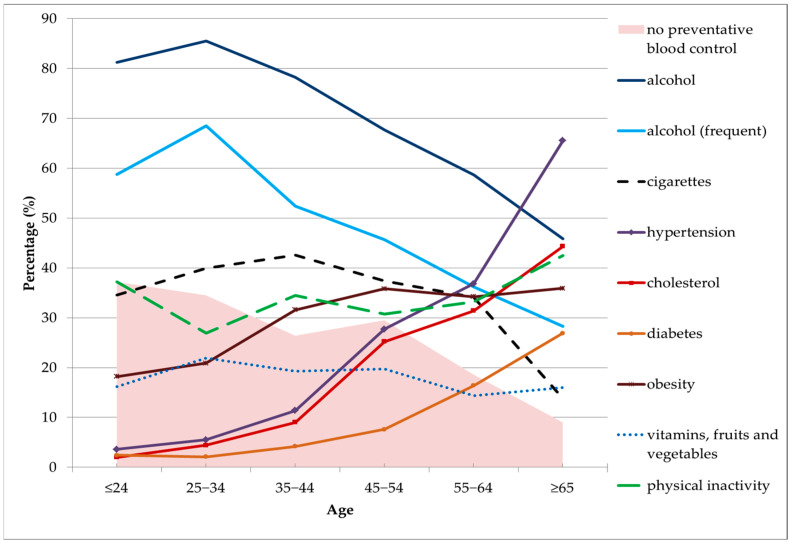
Prevalence of risk factors for NCDs in the Republic of Srpska by age (in %).

The average age of respondents with a prevalence of alcohol consumption was 34.1 years. The 25–34 age group had the highest prevalence of alcohol consumption. More than 80% of people who consumed alcohol at least once a year were registered in this contingent. A higher prevalence was also confirmed for the youngest population (≤24 years), as well as for the population aged 35–44 years. High values of frequent drinking (monthly or less) were also registered in the 35–44 age group. The lowest prevalence of alcohol consumption was noticed in the population aged 65 and above (Figure 1).

Among the risk factors related to smoking, the highest prevalence was recorded for persons aged 35–44 years (Figure 1). A high share of smokers were also in the age groups 25–34 and 45–54 years, as well as the youngest age group (≤24 years). The lowest smoking prevalence was noticed among the elderly (65 and above) (Figure 1).

A high percentage of the respondents were physically inactive, so physical inactivity was one of the most common risk factors for NCDs in this sample of the population of the RS. Of particular concern was the low level of physical activity in the young population (≤24 years), while the highest percentage of physically active persons was recorded in the age group 25–34 years. As expected, a low level of physical activity was registered for the old population (Figure 1).

The prevalence of alcohol consumption was particularly noticeable among older and middle-aged men, compared to women. Additionally, high differences were observed in physical inactivity, where the prevalence in young women was much higher than in men (Figure S4). Among the metabolic risk factors, the biggest differences according to gender were observed in the prevalence of hypertension and elevated cholesterol in the age group 65+ (Figure S5).

More than 90% of people aged 65 and above go to the doctor at least once a year. In contrast, less than 40% of respondents under the age of 24 do not attend preventive examinations at all (Figure 1). 

The binomial logistic regression analysis revealed that age, as a factor, had the highest impact on the intake of vitamins, fruits and vegetables, and the second highest impact on overweight and obesity (Table 5). At the same time, multinomial logistic regression showed that age was the dominant factor regarding the impact on all three metabolic characteristics (Table 6): blood pressure, cholesterol and blood sugar levels. From Table 7, which provides the results of the ordinal logistic regression, we can see that age, as a factor, had the highest impact on BMI and health control.

### 3.3. Type of Settlement and Risk Factors

The OR for type of settlement revealed higher alcohol consumption and smoking in the urban population, compared to the suburban and rural areas (Table 4).

A statistically significant difference was observed between the type of settlement and high blood pressure (Table 3). The observed differences were a consequence of the increased risk of hypertension in the countryside and pronounced risky behavior in urban areas. In the analysis of the type of settlement and cholesterol levels, blood sugar levels, overweight and obesity, physical activity and intake of vitamins, fruits and vegetables, statistically significant differences were not found (Table 3).

The binomial logistic regression showed that type of settlement, as a factor, had the highest impact on the intake of vitamins, fruits and vegetables, and health control, while it had the second highest impact on overweight and obesity (Table 5). Simultaneously, the multinomial logistic regression revealed that the type of settlement was the second most dominant factor regarding the impact on blood pressure level (Table 6).

### 3.4. Education and Risk Factors

The highest prevalence of risk factors was registered in respondents with a lower level of education, especially in regard to metabolic factors (Table 3). More than half of the lower-educated respondents had high blood pressure, while more than 30% had elevated cholesterol levels and increased blood sugar levels. Additionally, a significant number of the lower-educated persons were obese or overweight, as well as physically inactive. The highest prevalence of smoking was registered among respondents with secondary education. Respondents with higher education had higher odds of alcohol consumption by 30% compared to those with primary and secondary education (Table 4).

Statistical analysis showed a high degree of statistical significance between education levels and all variables except overweight and obesity, where no statistical significance was found (Table 3).

The binomial logistic regression revealed that education, as a factor, had the highest impact on smoking and tobacco use, alcohol consumption and blood pressure level (Table 5). Furthermore, it was the second most dominant factor regarding the impact on the blood sugar level, and according to the multinomial logistic regression, education was the second most dominant factor regarding the impact on the cholesterol level (Table 6). In Table 7, the results of the ordinal logistic regression are presented, indicating that education, as a factor, had the highest impact on the intake of vitamins, fruits and vegetables.

For the age group <35 years, the prediction accuracies of the binomial logistic regression for the blood pressure level, cholesterol level and blood sugar level were all above 95%. Unexpectedly, we found these results for the prediction accuracy to be strong. The most prominent factors for these target variables were gender and education level. Additionally, the second most prominent factors were working status and the type of settlement. However, one can rationalize these outcomes by observing that the younger population was much less prone to metabolic issues when compared to the older population (Table 5).

Regarding the age group 35≤ age <55, the prediction accuracy of the binomial logistic regression for high blood sugar (diabetes) was above 90% (almost 95%). The most prominent factor for diabetes (as a target variable) was the level of education, and the second most prominent was gender. The type of settlement was the most occurring prominent factor, which, interestingly, suggests a strong connection between demographic and medical variables. Furthermore, considering the high accuracy score in our regression analysis, education level emerged as a very important factor (Table 5).

The accuracy scores for the group ≥55 were significantly lower when compared to the previous two groups (age less than 35, and age between 35 and 54). The best prediction accuracy was obtained for health control (85.16%). The most prominent factor for this target variable seemed to be working status. The second most prominent was, according to our computations, education level (Table 5).

### 3.5. Working Status of Population and Risk Factors

Analysis of behavioral and metabolic risk factors and working status showed statistical significance for all tested variables, particularly between sociodemographic factors and metabolic risk factors (Table 3). The research in this paper shows that more than half of the retired respondents had high blood pressure, while a high proportion had high cholesterol and high blood sugar. Additionally, overweight and insufficient physical activity were risk factors for these age groups. 

Behavioral risk factors had a significantly lower prevalence in the retiree population. This is supported by the fact that less than 20% are smokers and consume alcohol at least once a week.

Due to the more favorable age structure, the student population is significantly less exposed to risk factors. Still, the prevalence of smokers among students and the prevalence of physically inactive students were high. Employed working status was associated with higher odds of smoking (20% higher risk), alcohol consumption (70.9%) and cholesterol level (21.7%), and lower odds of hypertension, diabetes, and overweight and obesity, as well as physical activity and consumption of vitamins, fruits and vegetables (Table 4).

The results of the binomial logistic regression show that working status, as a factor, did not exercise dominance (the most influential) in any of the cases. However, as we can see from Table 5, working status was the second most influential factor regarding: smoking and tobacco use, alcohol consumption, physical inactivity, cholesterol level and health control, while the multinomial logistic regression revealed that education is the second most dominant factor regarding the blood sugar level (Table 6). Additionally, the multinomial logistic regression on age groups did not yield any new different or substantial observations.

### 3.6. Income and Risk Factors

Based on the chi-square test, statistical significance (*p* < 0.001) was established between income and the levels of blood pressure, cholesterol and blood sugar, overweight and obesity, alcohol consumption and use of tobacco products (Table 3). Statistical testing revealed no differences between material status and physical inactivity. The biggest statistical differences were observed between material status and intake of vitamins, fruits and vegetables.

The binomial logistic regression found that income, as a factor, had the highest impact in the case of the cholesterol level (binary-viewed), as one can see from Table 5. This is the only case where income was a dominant factor, while in all others, regardless of whether it was a binomial, multinomial or ordinal analysis, income was not dominant. Further, it was neither the second most dominant factor, nor the least dominant factor, in any case. Somehow, it seems that the impact of income, as a factor, was somewhat average.

It seems that for the younger population, gender plays a very important role concerning their healthcare behavioral characteristics. However, the coefficients in Table 7 suggest that these characteristics in older people are more affected by their corresponding incomes.

## 4. Discussion

Differences in demographic and socioeconomic characteristics represent the main determinants of risk factors among the RS’s population. The results in this paper suggest a high impact of demographic determinants in relation to the behavioral and metabolic risk factors for the occurrence of the leading chronic diseases. The presence of metabolic factors was related to older population groups, especially the elderly, while the behavioral risk factors were much more common in the young and middle-aged populations. The prevalence of hypertension and high blood sugar was related to the male population, while the presence of cholesterol was more pronounced in women. 

Exposure to behavioral risk factors among the young and middle-aged populations was particularly pronounced for alcohol consumption and smoking. The total prevalence of smoking in this sample of the population of the RS was 36.2%. However, when occasional smokers are excluded, the proportion of permanent smokers (daily smokers) in this sample was at the level of 28.2%, which is among the highest values in Europe. According to Eurostat, 18.4% of the population in the EU are registered as daily smokers. The highest prevalence of smoking was registered in Bulgaria (28.7%), Greece (23.6%), Latvia (22.1%), Germany (21.9%) and Croatia (21.8%) [64]. Previous studies indicate that people with a lower level of education have a higher risk of smoking [65,66,67]. The results in this study indicate the reverse finding, with the highest prevalence of smoking among people with high school education. The lack of smokers with primary education is reflected in the fact that the largest percentage of this population is the elderly population. Regardless of some studies [66,68,69] that found that the frequency of smoking was much higher in men, the results obtained for the RS indicate smaller differences between genders. The most common smokers in the RS are middle-aged and young populations, while the number of smokers in the oldest cohort was significantly lower. In this context, the prevalence of anti-smoking among the younger age group and the formal education of students about the harmful effects of smoking should be integrated into all active anti-smoking programs. Alcohol is another lifestyle habit identified as a behavioral risk factor and an important public health issue. About 5.3% of all deaths in the world are the result of harmful alcohol consumption [70]. A study conducted in the Czech Republic proved that the prevalence of dangerous alcohol consumption in the adult population is between 16.8 and 17.6% [71]. Similar to previous studies [72], it was found that alcohol consumption in the RS is more pronounced in the male population, particularly in terms of frequent use. This was also confirmed in a study conducted by Cooper and Wilsnack [73,74]. The prevalence of alcohol consumption in the RS decreases with age, which is also confirmed in other populations [75]. Additionally, our study showed that the prevalence of alcohol consumption was higher among respondents in the city, while the most common consumers were people with higher and secondary education. One of the models of suppressing risk factors is to ban the sale of alcohol and tobacco to minors. In order to reduce NCD risk factors, the involvement of educational institutions could have an important role through diverse lectures and seminars. As one of the main public policy activities, it is necessary to better inform the population about the negative consequences of NCDs, particularly in the younger population.

Physical inactivity, healthy food consumption, and overweight and obesity are important behavioral risk factors that could also contribute to the risk of NCDs. Over 1/3 of adults in the EU (36.2%) are physically inactive [76]. Similar results were recorded among the population of Poland (35%) [77], while in Croatia, 30.5% of the population was registered as inactive [78]. Compared to the EU, the prevalence of physical inactivity in the RS is lower (33.4%). General physical inactivity is high in older age groups [76,79,80], and particularly among women [78,79], which was also found in the RS population, where the elderly were significantly less active in walking, jogging and running, with a higher percentage of physically inactive women compared to men. Our results indicate that more than half of the respondents had a poor or average intake of vitamins, fruits and vegetables. The results thus far indicate that this risk factor occurs more often in lower socioeconomic groups and the population with a lower income [81]. Accordingly, higher-quality nutrition in this study was recorded among the highly educated and the student population. Additionally, a healthier diet was registered in women, and a significantly lower intake of vitamins, fruits and vegetables was found in men, which agrees with previous research [82].

Overweight and obesity affect about 60% of the adult population of the European region [83]. According to data for 2016, the global percentage was 52% [84]. A high prevalence of obesity is also registered in the countries of the Western Balkans: for example, the prevalence of obesity in Serbia is 23.6%, while overweight is registered in 36.9% of the population, which is among the highest values in Europe [85]. Regarding the RS, almost half of the respondents had a problem with overweight and obesity. In contrast to the population of Poland, where the prevalence of overweight and obesity is similar for both genders [86], our research confirmed the higher vulnerability of men. 

The results in this study indicate that metabolic risk factors emphasize gender differences. High blood pressure and cholesterol are more frequent four to five years earlier in the male than the female population, while high glucose levels in females are recorded two years earlier than in males. According to the WHO, metabolic risk factors could contribute to the various metabolic changes leading to an increased risk of NCDs. About 22% of the population of the EU has high blood pressure (2019) [87]. The highest prevalence of hypertension was registered in Croatia in 2019 (37%), while the lowest values were found in the countries of Western Europe [87]. The prevalence of hypertension in the RS’s population is lower than in neighboring countries. The highest prevalence of hypertension is present in men [88]. However, in the elderly population, elevated blood pressure is more typical of women [89]. The distribution of respondents by gender indicates that men in the RS are more vulnerable than women.

The global prevalence of diabetes was estimated at 9.3% (463 million people) in 2019 [90]. Some projections until 2045 indicate an increase in the prevalence of diabetes worldwide to 10.9% (700 million) [41]. The results of our research show that the share of people with elevated blood sugar levels is 6.5%, which is lower than the EU average [91], with a higher prevalence of diabetes in the male population. The prevalence of diabetes is higher in men than in women [92,93], which was best illustrated in a study conducted in Portugal in which diabetes was registered in 14.2% of men and 9.5% of women [94].

The prevention and control of risk factors are fundamental determinants of the reduction in morbidity and mortality from NCDs [9,95]. Prevention in the RS can be described as unsatisfactory. Similar to previous studies [96], the majority of the RS population do not know (do not measure) the values of particular risk factors. Particularly alarming is the fact that a third of the population under 55 years does not check their blood count at all. Higher prevention awareness was observed in the female and elderly populations. The phenomenon that the population with a lower level of education performs health examinations more often is a consequence of the age structure, given that the majority of the population with this level of education is older. A similar example can be observed in the rural population, which is mainly represented by the elderly population. Unemployed people used blood analysis services less often, which we can place in the context of material position and socioeconomic differences.

The main strength of the findings in this study relates to their importance for formulating demographic policies as well as measures of population policies that are closely related to policies of public health. There has been an evident increase in mortality [83], especially from NCDs, which are directly related to behavioral and metabolic risk factors. The results in this study could contribute to the prevention and mitigation of the leading causes of these diseases.

Some limitations of this study also stand out. The main weakness is related to the lack of demographic and socioeconomic data in order to assess the impact of both behavioral and metabolic risk factors on the single causes of diseases among NCDs. Another limitation is the cross-sectional design of the study and the lack of causal ratio estimation. Additionally, the accuracy of self-reported risk factors could affect the measurement and dissimulate the results. The survey response about risk factors of NCDs depends on various factors related to the understanding of health problems, as well as willingness to report health issues. 

## 5. Conclusions

This is the first cross-sectional study of demographic and socioeconomic variables in relation to the behavioral and metabolic risk factors for NCDs in the RS. This study reveals the significant presence of risk factors among the RS population, with higher involvement of metabolic risk factors among the older population, while the prevalence of behavioral factors is related to younger age groups, especially in the case of alcohol consumption and smoking. This study makes a few meaningful contributions to the better understanding of NCD risk factors in the RS population. First, despite the fact that other studies confirm the relation between lower education and a higher risk of smoking, the results for the RS offer reverse findings, with higher odds of smoking among persons with higher education. Secondly, in regard to smoking, our results indicated lower odds between genders too. Thirdly, the prevalence of hypertension was found to be lower in the RS population compared to some neighboring countries. Finally, physical inactivity was found to be one of the most common risk factors of NCDs in the RS.

A reduction in NCD risk factors in the RS can be achieved through primary, as well as secondary and tertiary prevention. Simultaneously, the role of the government could be significantly enhanced through more frequent promotion of healthy lifestyles via the construction and landscaping of green areas, trim tracks, cycle paths and other facilities directed to the quality of life in the community.

## Figures and Tables

**Table 1:** Distribution of respondents according to their demographic and socioeconomic characteristics.

Variables	Type	Men		Women		Total	
		*n*	%	*n*	%	*n*	%
Gender		1062	46.0	1249	54.0	2311	100
Age	≤24 year	228	21.5	388	31.1	616	26.7
	25–34 year	271	25.5	253	20.3	524	22.7
	35–44 year	223	21.0	232	18.6	455	19.7
	45–54 year	168	15.8	161	12.9	329	14.2
	55–64 year	97	9.1	123	9.8	220	9.5
	65+	75	7.1	92	7.4	167	7.2
Type of settlement	Rural	164	15.5	202	16.2	366	15.8
	Suburban	195	18.4	264	21.1	459	19.9
	Urban	702	66.2	783	62.7	1485	64.3
Education level	Primary	44	4.1	74	5.9	118	5.1
	Secondary	638	60.1	693	55.5	1331	57.6
	High	380	35.8	481	38.5	861	37.3
Working status	Employed	655	61.7	630	50.5	1285	55.7
	Unemployed	156	14.7	182	14.6	338	14.6
	Students	157	14.8	327	26.2	484	21.0
	Pensioners	94	8.9	108	8.7	202	8.7
Income	Low	154	14.5	134	10.8	288	12.5
	Middle	525	49.5	644	51.8	1169	50.7
	High	381	35.9	466	37.5	847	36.8

**Table 2:** Results of descriptive statistics for the risk factors related to NCDs in the Republic of Srpska (RS).

Variables	Answer	Men		Woman		Total	
		*n*	%	*n*	%	*n*	%
Smoking and Tobacco Use	Daily smoker	319	48.9	333	51.1	652	28.8
	Non-daily smoker	71	38.4	114	61.6	185	8.0
	Never/ex-smoker	672	63.3	802	64.2	1474	63.8
Alcohol consumption	Yes	898	84.6	836	66.9	577	75.0
	No	164	15.4	413	33.1	1734	25.0
Frequent alcohol	Yes	727	68.5	507	40.8	1234	53.5
consumption	No	334	31.5	737	59.2	1071	46.5
Blood pressure level (≥140/90 mmHg)	Yes	193	18.2	193	15.5	386	16.7
	No	681	64.1	887	71.0	1568	67.8
	I do not know	188	17.7	169	13.5	357	15.4
Cholesterol level (≥5.0 mmol/L)	Yes	136	12.8	166	13.3	302	13.1
	No	658	62.0	817	65.4	1475	63.9
	I do not know	268	25.2	265	21.2	533	23.1
Blood sugar level (≥7.0 mmol/L)	Yes	77	7.3	74	5.9	151	6.5
	No	811	76.4	1011	80.9	1822	78.8
	I do not know	174	16.4	164	13.1	338	14.6
Overweight and obesity	BMI < 25.0	506	46.2	647	53.6	1153	50.1
	BMI 25.0–29.9	408	37.3	388	32.2	796	34.6
	BMI ≥ 30.0	181	16.5	171	14.2	352	15.3
Physical activity (>150 min/week)	Yes	774	72.9	765	61.2	1539	66.6
	No	288	27.1	484	38.8	772	33.4
Intake of vitamins, fruits and vegetables	Low	253	24.0	170	13.7	423	18.4
	Middle	422	40.0	470	37.8	892	38.9
	High	379	36.0	602	48.5	981	42.7
Health control (per year)	By no means	383	36.1	300	24.0	683	29.6
	Once	443	41.8	616	49.4	1059	45.9
	Twice	129	12.2	185	14.8	314	13.6
	Repeatedly	105	9.9	147	11.8	252	10.9

**Table 3:** Results of statistical analysis of demographic and socioeconomic characteristics and risk factors in a sample of the population of the RS.

Variables	Test	Gender	Age	Type of Settlement	Education Level	Working Status	Income
Smoking and Tobacco Use	χ^2^	0.217	170.970 *	10.953	15.464	42.902	10.516
	*p*	0.641	<0.001	0.004	<0.001	<0.001	0.005
Alcohol consumption	χ^2^	95.162	524.182 *	28.817	79.321	110.020	16.472
	*p*	<0.001	<0.001	<0.001	<0.001	<0.001	<0.001
Frequent alcohol	χ^2^	177.454	95.066 *	42.460	42.878	61.897	1.710
consumption	*p*	<0.001	<0.001	<0.001	<0.001	<0.001	0.425
Blood pressure level	χ^2^	13.029	482.641 **	12.626	180.250	393.122	50.258
	*p*	0.001	<0.001	0.013	<0.001	<0.001	<0.001
Cholesterol level	χ^2^	5.194	333.214 **	2.275	65.610	209.685	40.996
	*p*	0.075	<0.001	0.685	<0.001	<0.001	<0.001
Blood sugar level	χ^2^	7.225	157.654 **	1.638	55.778	136.777	29.522
	*p*	0.027	<0.001	0.802	<0.001	<0.001	<0.001
Overweight and obesity	χ^2^	9.972	58.026	0.294	6.883	46.422	6.000
	*p*	0.007	<0.001	0.990	0.142	<0.001	0.199
Physical inactivity	χ^2^	34.991	244.699 *	5.654	16.753	10.642	4.363
	*p*	<0.001	<0.001	0.059	<0.001	0.014	0.113
Intake of vitam., fruits and veget.	χ^2^	54.533	0.18	5.933	19.886	27.022	107.594
	*p*	<0.001	0.894	0.204	<0.001	<0.001	<0.001
Health control	χ^2^	40.289	141.606	15.225	132.584	243.840	29.905
	*p*	<0.001	<0.001	0.019	<0.001	<0.001	<0.001

* Wald test; ** Chi-square value (likelihood ratio test). Clarification for risk factors: Smoking and Tobacco Use (yes (including occasional smokers), no); Alcohol consumption (yes, no); Frequent alcohol consumption (yes, no); Blood pressure level (yes, no, I don’t know); Cholesterol level (yes, no, I don’t know); Blood sugar level (yes, no, I don’t know); Overweight and obesity (yes, no); Physical inactivity (yes, no); Intake of vitamins, fruits and vegetables (low, middle, high); Health control (by no means, once, twice, repeatedly).

**Table 4:** Odds ratios (ORs) of NCD risk factors associated with demographic and socioeconomic characteristics in a sample of the population of the RS.

Variables	Test	Gender	Age	Type of Settlement	Education Level	Working Status	Income
		Male/ Female	18–44/45+	Urban/ Suburb and Rural	Tertiary/ Primary and Secondary	Employment/Unemployment	High and Middle/Low
Smoking and Tobacco Use	OR	1.041	1.409	1.324	0.811	1.204	0.696
	95% CI	0.878–1.234	1.168–1.701	1.106–1.585	0.679–0.968	0.939–1.542	0.542–0.894
Alcohol consumption	OR	2.705	2.980	1.622	1.300	1.709	1.704
	95% CI	2.206–3.317	2.449–3.627	1.338–1.967	1.066–1.586	1.311–2.228	1.309–2.219
Frequent alcohol consumption	OR	3.164	2.383	1.721	1.204	1.298	1.171
	95% CI	2.664–3.758	1.989–2.856	1.449–2.044	1.016–1.427	1.021–1.651	0.914–1.499
Blood pressure level	OR	1.302	0.114	0.732	0.597	0.839	0.553
	95% CI	1.042–1.628	0.088–0.146	0.583–0.920	0.470–0.759	0.600–1.174	0.413–0.742
Cholesterol level	OR	1.017	0.114	0.882	0.981	1.217	0.532
	95% CI	0.793–1.305	0.086–0.152	0.682–1.140	0.761–1.264	0.821–1.805	0.388–0.730
Blood sugar level	OR	1.297	0.175	0.945	0.657	0.926	0.480
	95% CI	0.931–1.808	0.122–0.252	0.669–1.335	0.458–0.943	0.548–1.565	0.320–0.722
Overw. and obesity (BMI ≥ 25/<25)	OR	1.3473	0.544	0.996	0.938	0.909	0.678
	95% CI	1.143–1.588	0.448–0.659	0.822–1.207	0.775–1.136	0.700–1.181	0.520–0.883
Physical inactivity	OR	0.588	0.949	0.879	0.714	0.960	0.763
	95% CI	0.493–0.702	0.787–1.143	0.735–1.052	0.595–0.857	0.743–1.241	0.592–0.984
Vitam., frui. and veg. (Low/mid. and high)	OR	1.992	1.128	0.893	0.630	0.688	0.370
	95% CI	1.607–2.469	0.894–1.423	0.718–1.111	0.500–0.793	0.518–0.914	0.282–0.486
Health control	OR	0.560	0.546	1.197	1.729	1.628	1.761
	95% CI	0.468–0.671	0.444–0.672	0.995–1.440	1.425–2.098	1.268–2.090	1.366–2.272

**Table 5:** Prediction accuracy and exponentiated coefficients of binomial logistic regression by age, based on demographic and socioeconomic characteristics and risk factors in a sample of the population of the RS.

Grouping	Variables	Accuracy Score	Gender	Age	Type of Settlement	Education Level	Working Status	Income
All ages	Smoking and Tobacco Use (bin)	64.85%	0.879	1.141	1.271	1.676 *	1.592 **	1.187
	Alcohol consumption (bin)	77.40%	0.333	0.715	0.709	1.310 *	0.926 **	1.097
	Physical inactivity (bin)	66.06%	1.554 *	1.023	1.016	0.678	1.017 **	0.993
	Blood pressure level (bin)	85.27%	1.191 **	0.464	0.909	1.573 *	1.003	1.182
	Cholesterol level (bin)	86.73%	1.025	0.467	1.002	0.957	1.075 **	1.225 *
	Blood sugar level (bin)	93.59%	1.295 *	0.539	1.067	1.210 **	0.951	1.136
	Overweight and obesity (bin)	73.11%	0.782	1.278 *	1.076 **	0.917	0.852	0.828
	Vitamins, fruits and veg. (bin)	81.49%	0.527	0.910 **	0.983 *	0.622	0.780	0.526
	Health control (bin)	70.67%	0.595	0.733	1.029 *	0.675	0.841 **	0.693
<35 age	Smoking and Tobacco Use (bin)	63.44%	1.015	1.192	1.438	1.558 *	1.467 **	1.321
	Alcohol consumption (bin)	83.22%	0.715	0.994	0.798	1.119 *	0.855	1.108 **
	Physical inactivity (bin)	67.64%	2.262 *	0.730	1.047 **	0.601	0.858	0.874
	Blood pressure level (bin)	95.42%	1.190	0.836	0.893	1.966 *	1.652 **	1.403
	Cholesterol level (bin)	96.82%	1.078 *	0.523	0.928 **	0.798	0.865	0.662
	Blood sugar level (bin)	97.81%	0.557	0.882	1.338 **	1.622 *	0.979	1.154
	Overweight and obesity (bin)	80.47%	0.732	0.917 **	1.028 *	0.744	0.747	0.829
	Vitamins, fruits and veg. (bin)	80.81%	0.617	0.978 *	0.876 **	0.684	0.766	0.699
	Health control (bin)	64.14%	0.475	0.820	1.169 *	0.819	0.942 **	0.893
35≤ age <55	Smoking and Tobacco Use (bin)	58.69%	0.831	1.186	1.092	1.664 *	1.328 **	1.217
	Alcohol consumption (bin)	75.8%	0.208	0.655	0.755	1.435 **	0.826	0.909 **
	Physical inactivity (bin)	67.18%	1.297 *	0.675	1.011	0.765	0.850	1.032 **
	Blood pressure level (bin)	81.77%	1.496 *	0.436	0.824	1.131 **	0.888	1.063
	Cholesterol level (bin)	84.26%	1.313 *	0.334	0.811	0.833	1.176 **	1.164
	Blood sugar level (bin)	94.42%	1.405 **	0.596	1.094	1.954 *	1.178	0.889
	Overweight and obesity (bin)	66.14%	0.736	1.143 **	0.881	0.903	1.171 *	1.101
	Vitamins, fruits and veg. (bin)	80.26%	0.682	0.913	0.970 **	0.699	1.148 *	0.597
	Health control (bin)	71.91%	0.715	1.076 *	1.009 **	0.557	0.902	0.609
≥55 age	Smoking and Tobacco Use (bin)	75.21%	1.166	2.803 *	1.103	1.345	1.158	1.706 **
	Alcohol consumption (bin)	66.94%	0.174	0.950	0.827	1.183 **	0.885	1.579 *
	Physical inactivity (bin)	61.63%	1.025 **	1.002	0.938	0.810	1.095 *	0.995
	Blood pressure level (bin)	63.37%	0.770	0.467	0.856	1.659 *	0.889	1.053 **
	Cholesterol level (bin)	61.90%	0.642	0.924	1.046 **	0.877	0.874	1.333 *
	Blood sugar level (bin)	79.07%	0.853	0.724	1.261 *	0.887	1.015 **	0.569
	Overweight and obesity (bin)	63.72%	0.782	1.278 *	1.076 **	0.917	0.852	0.828
	Vitamins, fruits and veg. (bin)	84.17%	0.264	1.230 *	1.096 **	0.388	0.707	0.277
	Health control (bin)	85.16%	0.501	0.503	0.599	0.735 **	0.841 *	0.609

The largest and second largest values among coefficients in each row are denoted with one and two asterisks (*), respectively; (bin) = binary; Vitamins, fruits and veg. = Intake of vitamins, fruits and vegetables.

**Table 6:** Prediction accuracy and exponentiated coefficients of multinomial logistic regression by age, regarding the demographic and socioeconomic characteristics and risk factors in a sample of the population of the RS.

Grouping	Variables	Accuracy Score	Gender	Age	Type of Settlement	Education Level	Working Status	Income
All ages	Blood pressure level	70.04%	0.859	1.717 *	1.187 **	0.847	0.924	1.029
	Cholesterol level	63.92%	1.040	1.744 *	1.119	1.171 **	0.917	1.018
	Blood sugar level	78.75%	0.911	1.505 *	0.987	0.978	1.106 **	0.947
<35 age	Blood pressure level	74.79%	0.772	1.062 *	1.008 **	0.619	0.689	0.917
	Cholesterol level	69.34%	1.039	1.896 *	1.067 **	1.048	0.975	0.921
	Blood sugar level	81.14%	1.187 *	0.749	0.987 **	0.697	0.911	0.975
35≤ age <55	Blood pressure level	69.01%	0.916	1.667 *	1.291 **	0.908	0.966	1.017
	Cholesterol level	63.92%	0.859	2.168 *	1.157	1.164 **	0.937	0.934
	Blood sugar level	79.89%	0.876	1.419 *	1.018 **	0.892	0.971	0.965
≥55 age	Blood pressure level	57.5%	1.194	1.811 *	1.096	1.080	1.264 **	1.247
	Cholesterol level	49.48%	1.572 *	1.372 **	1.121	1.082	1.017	0.938
	Blood sugar level	69.91%	0.740	1.409 *	1.109 **	0.866	1.047	0.744

The largest and second largest values among coefficients in each row are denoted with one and two asterisks (*), respectively.

**Table 7:** Exponentiated coefficients of ordinal logistic regression by age, regarding the demographic and health characteristics and risk factors in a sample of the population of the RS.

Grouping	Variables	Gender	Age	Type of Settlement	Education Level	Working Status	Income
All ages	Overweight and obesity	0.806	1.248 *	1.007	0.824	0.841	0.837
	Vitamins, fruits and veg.	1.689	1.040	0.953	1.405 *	1.237	1.804
	Health control	1.528	1.374 *	0.974	1.108	1.296	1.387
<35 age	Overweight and obesity	0.769	0.947 **	1.051 *	0.727	0.743	0.872
	Vitamins, fruits and veg.	1.606 *	1.025	0.978	1.518 **	1.353	1.487
	Health control	1.804 *	0.967	0.874	1.259 **	1.036	1.214
35≤ age <55	Overweight and obesity	0.752	1.210 *	0.868	0.948	1.190 **	1.023
	Vitamins, fruits and veg.	1.807 **	1.261	0.925	1.209	1.036	1.985 *
	Health control	1.431 **	1.205	0.933	1.294	1.185	1.530 *
≥55 age	Overweight and obesity	1.034 **	0.911	1.120 *	0.857	0.947	0.574
	Vitamins, fruits and veg.	1.596 **	1.476	0.896	1.541	1.111	2.586 *
	Health control	1.160	2.486 *	1.317	0.775	1.200	1.716 **

The largest and second largest values among coefficients in each row are denoted with one and two asterisks (*), respectively; Vitamins, fruits and veg. = Intake of vitamins, fruits and vegetables.

## Data Availability

The data presented in this study are available on request from the corresponding author.

## Supplementary Materials

The following supporting information can be downloaded at: https://www.mdpi.com/article/10.3390/healthcare11040483/s1, Figure S1: Cramer’s V correlation coefficients regarding the association between demographic and socio-economic variables and risk factors; Figure S2: A typical graph used in the elbow method concerning our data set. The x-axis represents the number of clusters, and the *y*-axis represents the cost of doing a *k*-modes clustering for a given number of clusters. The elbow is denoted with the red triangle; Figure S3: Histogram of individuals regarding (a) hypertension, (b) cholesterol, and (c) diabetes, across given clusters. The number of individuals without hypertension, cholesterol and diabetes is dominant in each cluster; Figure S4: Prevalence of behavioural risk factors for NCDs in the Republic of Srpska (RS) by gender and age; Figure S5: Prevalence of metabolic risk factors for NCDs in the RS by gender and age.
