# Supplementary material for: Behavioral and Metabolic Risk Factors for Noncommunicable Diseases among Population in the Republic of Srpska (Bosnia and Herzegovina)
Source: Healthcare (Basel). 2023 Feb 7;11(4):483. doi: 10.3390/healthcare11040483 (PMC9957477; doi:10.3390/healthcare11040483)
Supplement: Supplementary file 1 [file healthcare-11-00483-s001.zip › healthcare-2109522-supplementary.pdf]

Supplementary Materials

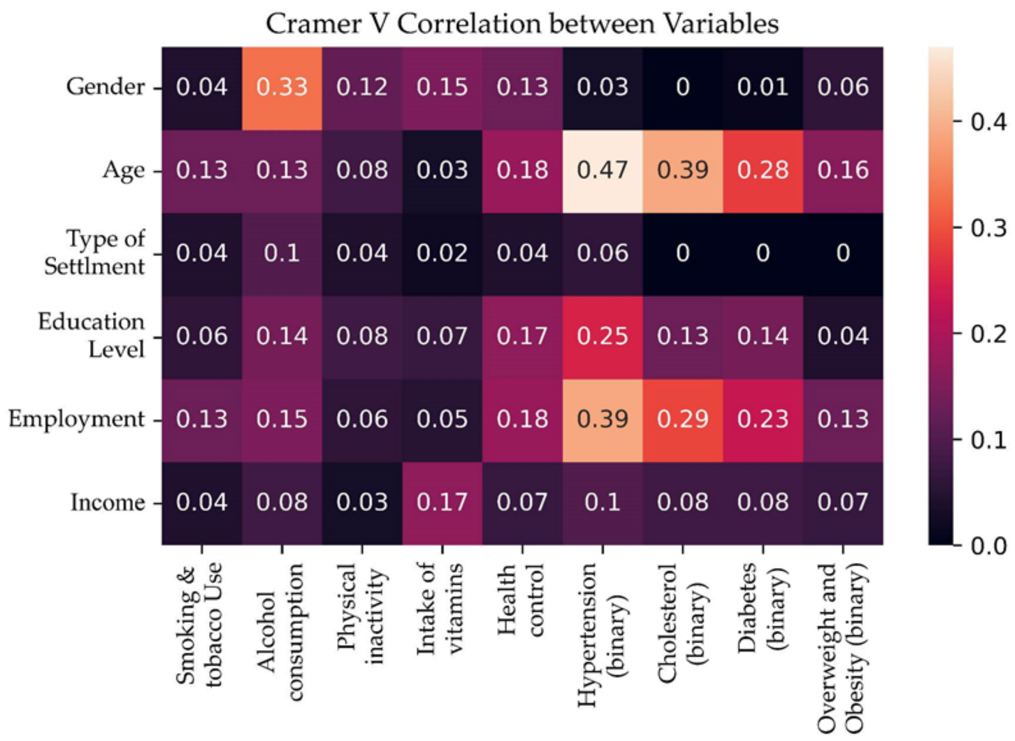

Figure S1: Cramer's V correlation coefficients regarding the association between demographic and socioeconomic variables and risk factors in a sample of the population of the Republic of Srpska (RS)

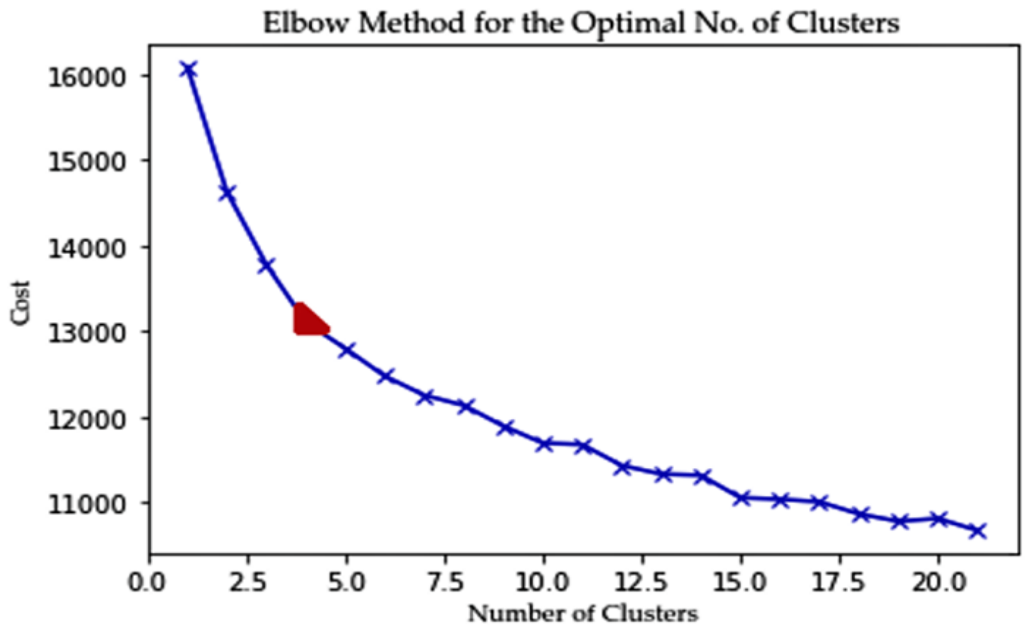

Figure S2: A tipical graph used in the elbow method concerning our data set. The x-axis represent the number of clusters, and the y-axis represent the cost of doing a k-modes clustering for a given number of clusters. The elbow is denoted with the red triangle

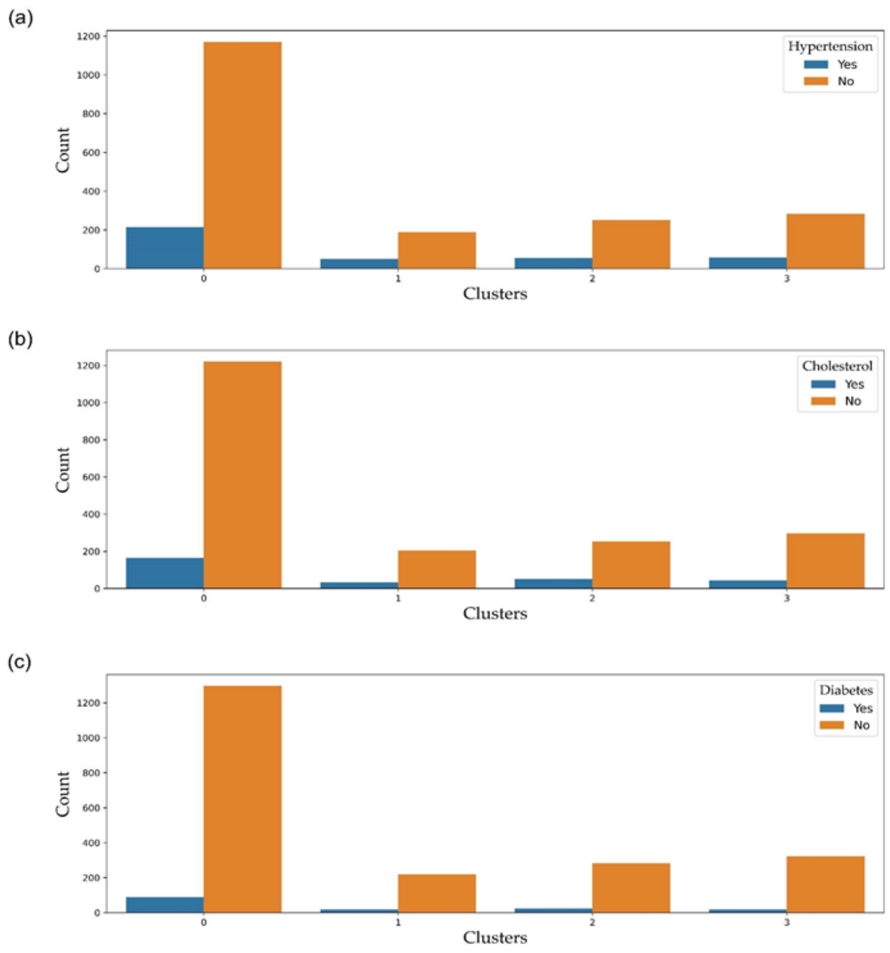

Figure S3: Histogram of individuals regarding a) hypertension, b) cholesterol and c) diabetes, across given clusters. The number of individuals without hypertension, cholesterol and diabetes is dominant in each cluster.

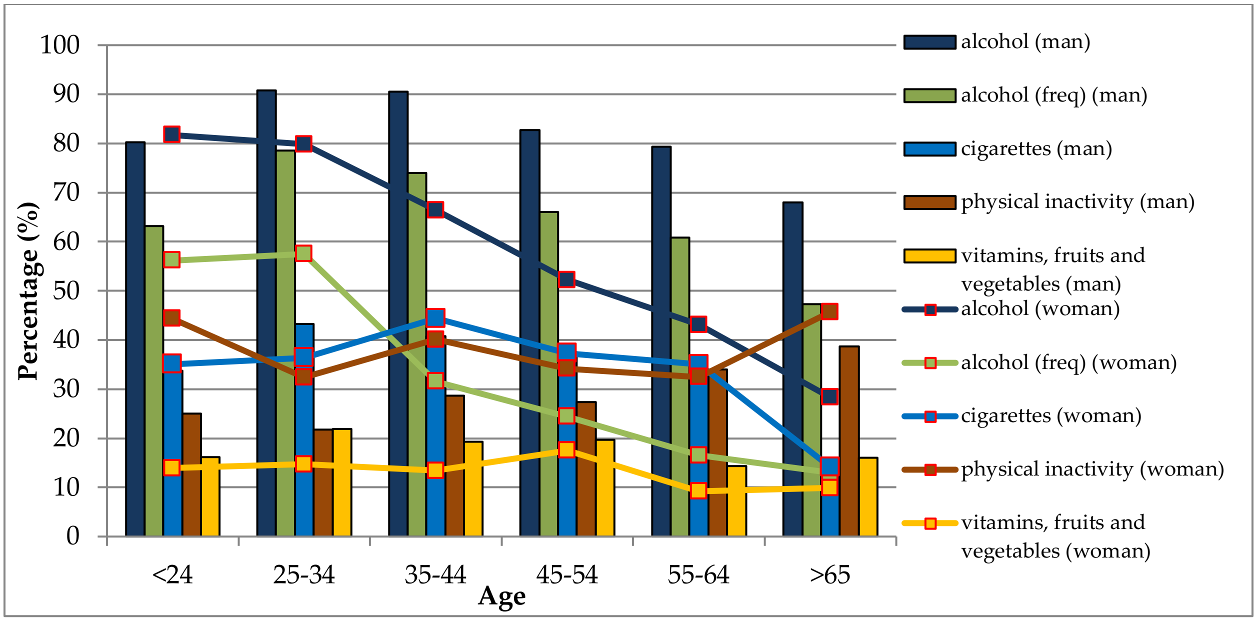

Figure S4: Prevalence of behavioral risk factors for Noncommunicable diseases (NCDs) in the RS by the gender and age

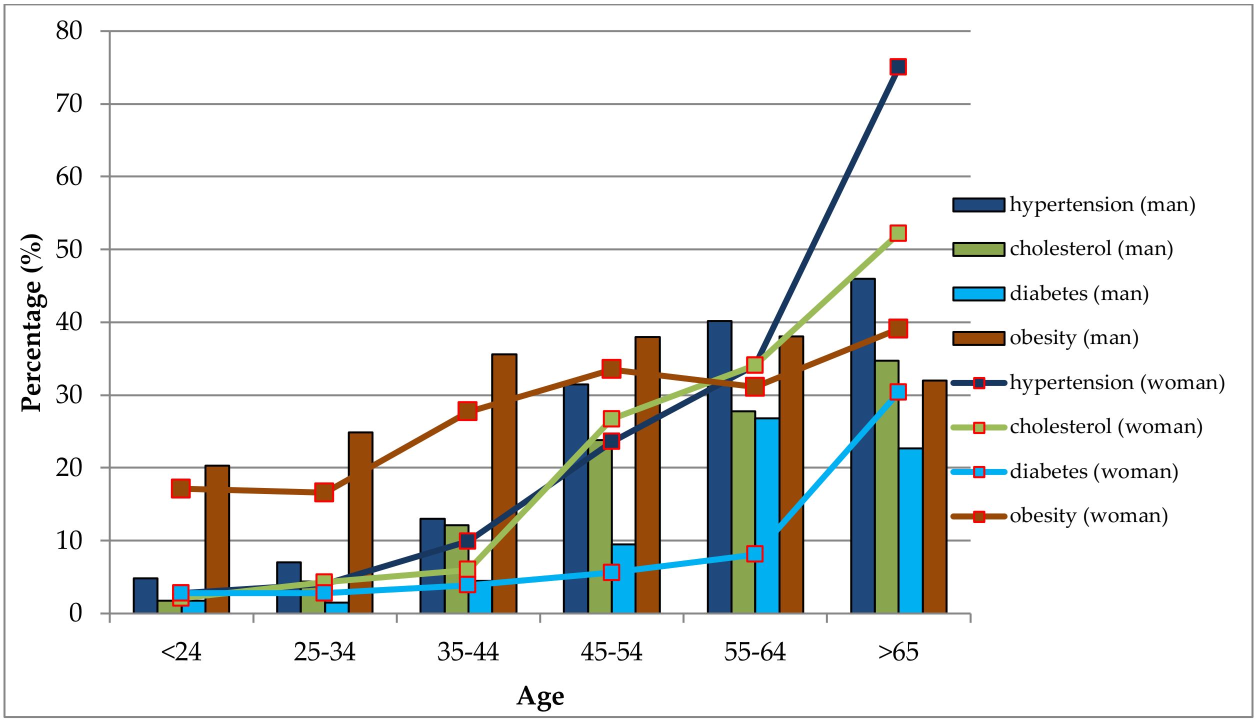

Figure S5: Prevalence of metabolic risk factors for Noncommunicable diseases (NCDs) in the RS by the gender and age
